# Individual Popularity, Peer Group Popularity Composition and Adolescents’ Alcohol Consumption

**DOI:** 10.1007/s10964-016-0611-2

**Published:** 2016-11-15

**Authors:** Rob Gommans, Christoph M. Müller, Gonneke W. J. M. Stevens, Antonius H. N. Cillessen, Tom F. M. Ter Bogt

**Affiliations:** 1 Centre for Child and Adolescent Studies, Utrecht University, Utrecht, The Netherlands; 2 Behavioural Science Institute, Radboud University, Nijmegen, The Netherlands; 3 Institute of Special Education, University of Fribourg, Fribourg, Switzerland

**Keywords:** Popularity, Alcohol use, Adolescence, Classroom composition, Social comparison

## Abstract

Previous studies have convincingly shown associations between popularity and adolescent drinking. This study examined whether the popularity composition of the peer group and the relative difference in popularity between adolescents and their peers are also associated with adolescent drinking. Participants were 800 adolescents (*M*_age_ = 14.73; SD_age_ = 1.00; 51.6 % girls) from 31 classrooms who completed peer ratings of popularity and self-reports of alcohol consumption. Results showed that drinking was higher among popular than unpopular adolescents, higher among popular adolescents surrounded by less popular classmates, and lower in classrooms with more variability in popularity. Thus, beyond individual popularity, peer group popularity composition also should be taken into account when investigating antisocial and health risk behaviors in adolescence such as drinking.

## Introduction

In adolescence, the peer group is highly salient (Brechwald and Prinstein 2011; Ryan 2001). During this period, interpersonal behaviors become more sophisticated and there is an increased awareness of social roles. Peer networks become larger and—in addition to the primary classroom context—start to include a larger array of peers both within school and outside of school. Due to these changes, more frequent and complex relationships with peers develop (Brown 1990). These increases in engagement with peers give adolescents the opportunity to compare themselves to others and to learn how they are doing—how similar or “successful” they are—compared to others (Festinger 1954; Piehler 2011).

One important dimension of the adolescent peer group is popularity. Popularity is peer-perceived and reflects prestige, visibility, and reputation (Cillessen and Marks 2011). Popularity is more salient in early adolescence than in other age groups (Cillessen and Rose 2005; LaFontana and Cillessen 2010). This is not surprising, because being popular in adolescence is correlated with enhanced self-perceptions, increased belongingness to the peer group, lower chances of rejection and exclusion, and access to valuable resources (Cillessen et al. 2011). Consequently, adolescents are motivated to engage in popularity-enhancing behaviors (such as aggression and substance use) and abstain from popularity-diminishing behaviors in order to acquire or maintain popularity (Caravita and Cillessen 2011; Cillessen et al. 2014; LaFontana and Cillessen 2010).

One important correlate of adolescent popularity is alcohol use. Studies have found consistent links between popularity and alcohol use (e.g., Ali et al. 2014; Balsa et al. 2011; Hubers et al. 2016; Mayeux et al. 2008; Tucker et al. 2011): Being more popular is associated with higher alcohol consumption. This link is important, given wide-spread public health concerns about alcohol use among youth (Cuijpers 2002; WHO 2014). However, focusing only on the association between adolescents’ own popularity and their drinking behavior ignores the context in which it occurs. Because group characteristics affect peer influence processes (Hartup 2005), alcohol consumption may depend not only on adolescents’ own popularity but also on the popularity status of the others in their group. So far, to our knowledge, no studies have examined the roles of individual status, peer group status, and their interaction, in the prediction of adolescent alcohol use. The goal of this article was to examine these three perspectives, individual, peer group, and individual-group interaction, with respect to the role of popularity in adolescent drinking. The classroom was used as the primary peer group, given previous findings indicating that the classroom as a social group is relevant for adolescent drinking (e.g., Gommans et al. 2016; Müller et al. 2015).

### Individual Popularity and Alcohol Consumption

At some point in adolescence, alcohol becomes a sign of adulthood and thus acquires reputational salience (e.g., Engels 2003). Despite the many negative effects of (heavy) drinking on adolescents, they see alcohol as a symbol of adult privilege and maturity and thus as something that will make them popular (Harton and Latané 1997; Moffitt 1993). Thus, there are associations between adolescents’ own popularity and their own drinking behaviors because adolescents see alcohol as something that enhances their status.

### Peers’ Popularity and Alcohol Consumption

Conversely, adolescents who are popular also influence others to drink. This is the domain of peer influence. Indeed, research has shown that popular adolescents play an important role in influencing others to drink (e.g., Bosari and Carey 2001; Bot et al. 2005; Chassin et al. 2009; Teunissen et al. 2012). This phenomenon is often explained by referring to mechanisms of *social learning*, such as peer contagion (e.g., Dishion et al. 2000; Fujimoto and Valente 2012), and imitation and modeling (e.g., Larsen et al. 2010). It is also explained by the desire for adolescents who are not popular to become popular or become friends with popular others (e.g., Cialdini and Richardson 1980; Dijkstra et al. 2010; LaFontana and Cillessen 2010). Thus by imitating the drinking behaviors of popular peers, adolescents expect to gain popularity themselves. There may be two motives for wanting popularity. One is the privileges of popularity itself, such as influence over others, control over social norms, and access to desirable resources (Moffitt 1993; Sandstrom 2011). The other is fear of and the desire to avoid the negative consequences of being unpopular (e.g., rejection, exclusion, negative evaluation; Bosari and Carey 2001; Schroeder and Prentice 1998).

### Group-Level Popularity and Alcohol Consumption

While the previous two perspectives are individual, the third presented is that of the group. It is also possible to examine adolescents’ drinking in the context of the group in which they interact. Kanter (1977) already stated that “groups with varying proportions of people of different social types differ qualitatively in dynamics and process” (pp. 965–966). Translating this notion to popularity and drinking, the status composition of a peer group in terms of popularity may significantly affect the dynamics of the group with respect to status-enhancing behaviors such as drinking. In other words, the popularity composition of the group may correlate significantly with individual drinking. However, to our knowledge, no studies have investigated this group perspective on adolescent drinking. Given that popularity is a ranking indicating one’s “success” relative to peers (Bukowski 2011) and that social comparison is a strong mechanism of peer influence in adolescence, the popularity composition of the group and the similarities and differences in popularity between adolescents and their peers may be associated with the occurrence of status-enhancing behaviors such as adolescent drinking.

## The Current Study

This study investigated whether adolescent alcohol consumption varied as a function of adolescents’ own popularity (Perspective 1), the popularity of others in their classroom (Perspective 2), and the comparison between own and others’ popularity and the heterogeneity of the others’ popularity (Perspective 3). Perspective 1 states that adolescents’ alcohol consumption is associated with their own popularity (*intrapersonal influence*; Ervin and Bonito 2014). Perspectives 2 and 3 state that adolescent drinking is associated with the popularity of the others, the variability in popularity among the others, and the relative comparisons in terms of status between adolescents and their primary peers *(interpersonal influence*). This study examined these perspectives together testing the following four specific hypotheses.

First, drawing on the already established association between popularity and alcohol consumption, we expected a positive association between adolescents’ own popularity and their own alcohol consumption (Hypothesis 1). This is an *actor effect*,^1^ that is, more popular adolescents were expected to drink more alcohol than less popular adolescents.

Second, we expected that popular adolescents influence others to drink. Thus, if there were more popular adolescents in a classroom, we expected the members of that classroom to drink more alcohol (Hypothesis 2). This is an *others’ effect*, that is, we expected adolescents to drink more alcohol in classrooms with more popular peers, or with a higher average level of popularity. This expectation was based on the reputational salience of drinking alcohol. We expected that adolescents drink more in classrooms where others are relatively popular because they want to increase their own popularity (e.g., LaFontana and Cillessen 2010) and want to avoid rejection and exclusion (e.g., Bosari and Carey 2001). Our expectation was also based on the principles of social learning (Brechwald and Prinstein 2011; Piehler 2011): When there is more drinking in a classroom because there are more popular peers, adolescents are exposed more to drinking and more likely to start drinking themselves through modeling and imitation (e.g., Fujimoto and Valente 2012; Teunissen et al. 2012).

Third, social comparison theory (Festinger 1954) states that self-evaluations and subsequent behaviors vary as a function of how one’s own characteristics relate to those of the group. That is, drinking may vary as a function of how popular an adolescent is compared to the average popularity of all others (i.e., the contrast between *actor effect* and *others’ effect*). Individual alcohol consumption may be highest when it allows adolescents to set themselves apart from all others, thereby strengthening their feelings of being and acting as an adult in contrast to all others. Thus, we expected that adolescents would drink more alcohol when they were popular in a classroom with unpopular others (Hypothesis 3).

Fourth, alcohol consumption also may depend on the heterogeneity of popularity in a classroom (an *others’ dissimilarity effect*). Drawing on self-categorization theory (Turner et al. 1987), in popularity-heterogeneous groups it is more likely that adolescents view themselves as different from their classmates than in popularity-homogeneous groups (Hogg 1993). As a consequence, group identification and cohesion will be lower in popularity-heterogeneous classrooms than in popularity-homogeneous classrooms (Hogg 1993). This would result in less conformity to the group (Aronson 2011; Cialdini and Trost 1998). As such, group diversity is a potential threat to the maintenance of group norms (e.g., related to alcohol; Feldman 2003). Previous research indeed has shown a positive association between drinking behavior and group cohesiveness (Kreager et al. 2011; Neighbors et al. 2010).

The same expectation can be based on the notion of an *ingroup prototype*. The ingroup prototype consists of the perceptions, attitudes, feelings, and behaviors that define group membership (Tindale et al. 2001). In a homogeneous group, it is very clear what the ingroup prototype is and what behaviors are needed to be a group member. In a heterogeneous group, this is less clear. Thus, in a popularity-heterogeneous group, it may be less clear for adolescents whether drinking will help them to be more popular or not. In other words, alcohol consumption may be less a prototypical behavior that determines one’s social position in popularity-heterogeneous groups than in popularity-homogeneous groups, and consequently the pressure to drink may be lower. This would also lead to the expectation that adolescents were less likely to drink when their classroom was more heterogeneous in popularity (Hypothesis 4).

## Method

### Participants and Procedure

The initial sample consisted of 800 13- to 16-year-old adolescents (*M*_age_ = 14.73; SD_age_ = 1.00; 51.6 % girls) from one secondary school in The Netherlands. Participants were in 31 classrooms in Grade 8 (*n* = 260), Grade 9 (*n* = 293), and Grade 10 (*n* = 247). Most participants were of native Dutch origin (91.8 %, *n* = 734). The Dutch school system is strongly classroom-oriented; most of the time at school students are with the same classmates and the classroom composition does not change much throughout the day or week. Consent procedures required by school policies were followed: Only adolescents who volunteered to participate and whose parents did not object to their participation were included. The response rate within classrooms was 95.3 % on average with illness as the main reason for non-participation. Participants with missing data (*n* = 38) or extreme outcome scores (*n* = 14) were excluded from the analyses resulting in a final sample of 748 adolescents.

Participants completed a computerized questionnaire programmed in InQuisit (2012) during a 60-min classroom session. At the beginning of the assessment, students were informed about the study and how to complete the questionnaires. Students were informed that the goal of the study was to assess their peer relationships and well-being at school. The confidentiality and privacy of their answers were emphasized. The students sat in a test-arrangement at their assigned desk, with adequate space between desks and screens placed on both sides of each laptop to guarantee privacy. At least two researchers were present to answer questions and to make sure instructions were followed.

### Measures

#### Alcohol consumption

Participants were asked to indicate on how many days in the last 30 days they consumed alcohol using a 7-point ordinal scale (0 = never, 1 = 1–2 days, 2 = 3–5 days, 3 = 6–9 days, 4 = 10–19 days, 5 = 20–29 days, 6 = all 30 days). Participants who indicated to have drunk alcohol on one or more days in the last 30 days were also asked to indicate how many glasses of alcohol they typically consumed on a given drinking day using a 10-point ordinal scale (0 = I never drink alcohol, 1 = less than 1 glass, 2 = 1 glass per day, 3 = 2 glasses per day, 4 = 3 glasses per day, 5 = 4 glasses per day, 6 = 5 glasses per day, 7 = 6 glasses per day, 8 = 7–10 glasses per day, 9 = more than 10 glasses per day). A Quantity-Frequency (QF) measure was calculated by taking the product of both scores (i.e., number of days times number of glasses). A higher score indicated more alcohol consumption. Participants with extreme scores of more than four standard deviations above zero (1.7 %, *n* = 14) were excluded from the analyses. An additional 38 participants (4.7 %) were excluded from the analyses because of missing data on either of the above two questions. The remaining 748 participants were included in all analyses even if they reported not to have drank alcohol in the last 30 days.

#### Popularity

Participants rated the popularity of every classmate (“In your class, how popular is…”) on a 6-point interval scale ranging from −3 to +3, with labels only at the endpoints (−3 = very unpopular, +3 = very popular). For each participant, the average popularity rating received from all classmates was computed (Cillessen 2009; Cillessen and Marks 2011).

### Analysis Strategy

The four hypotheses were tested with the *Group Actor-Partner Interdependence Model* (GAPIM; Garcia et al. 2015; Kenny and Garcia 2012). This is a new methodological framework that allows for the simultaneous modeling and analysis of complex relationships between individual and group characteristics.

The association between the average popularity rating received from classmates and individual alcohol behavior was the a*ctor effect* (*X*). For the *others’ effect* (*X′*), the general approach would be to regress individual alcohol consumption on the group mean of popularity. However, this would not control for the fact that the focal individual’s popularity is correlated with group popularity (i.e., the individual is also a member of the group; Kenny et al. 2002). Therefore, to avoid biased estimates of group composition, the *others’ effect* (*X′*) was calculated by taking the mean in popularity of the others in the classroom excluding the focal individual.

To calculate the *others’ dissimilarity effect* (*I′*), we extended Garcia et al. (2015) approach for dichotomous data to continuous data, since popularity was a continuous score. In the dichotomous case, the predictor score for two persons A and B is effect coded (e.g., gender: −1 = male, +1 = female). In that case, the product of the predictor scores of A and B is a measure of similarity, i.e., +1 if both are similar in gender; −1 if both are dissimilar in gender. This measure of similarity would be calculated for every possible dyad in the group, resulting in an average measure of similarity among all group members.

This approach does not work for a continuous score. For example, assume that popularity has three values: −1 (low), 0 (average) and +1 (high). The product of the popularity scores of two persons would indeed be −1 if both are most dissimilar (e.g., a − 1 and a + 1). However, when A and B are similar, the product term would correctly equal +1 when both are low (−1) or high (+1), but would incorrectly be 0 if both are average (0). In fact, the product would equal zero if either A or B was average (0). Recoding popularity to a 1–6 scale would not solve the problem as it would lead to multicollinearity, and subsequent grand mean centering to eliminate multicollinearity would repeat the original problem. As a solution, the *others’ dissimilarity* (*I′*) *effect* was computed by taking the standard deviation of popularity of the *n*–1 others in the classroom multiplied by −1 for ease of interpretation: −1 * √[ns^2^–*I*_i_^2^/*n*–2], where *s* ^2^ is the within-classroom variance. A score of zero then indicates exact similarity and increasing negative values indicate increasing dissimilarity.

A two-level multilevel approach was used (students nested within classrooms). Multilevel modeling was warranted based on an intraclass correlation (ICC) of .051 and a design effect (DE) estimate of 2.22 for alcohol consumption. All effects were included in the multilevel model as Level 1 predictors. The first step of computing an *others’ effect* (*X′*) and an *others’ dissimilarity effect* (*I′*) was done in SPSS 21. Subsequent model estimation was conducted in Mplus 7 (Muthén and Muthén 2012). All models were estimated using maximum likelihood with robust standard errors.

#### Model estimation and selection

Model estimation was done using a progressive strategy: From the simplest model in the first step to increasingly more complex models in subsequent steps. Participant age was included as a covariate in all models. First, an *empty model* with the *actor effect* (*X*), *others’ effect* (*X′*), and *others’ dissimilarity effect* (*I′*) constrained to zero was estimated to obtain a baseline for the sample-adjusted Bayesian information criterion (SABIC) as a measure of fit. Second, a *main effects model* was estimated including the two main effects—*actor effect* (*X*) and *others’ effect* (*X′*)—with the *others’ dissimilarity effect* (*I′*) constrained to zero. The fit of this model was compared with the *empty model*, where a smaller SABIC would indicate better fit. Third, we estimated the *complete model* with all three GAPIM terms—*actor effect* (*X*)*, others’ effect* (*X′*)*,* and *others’ dissimilarity effect* (*I′*)—and compared the fit of this model to the *main effects model*. The equation for the *complete model* for an individual outcome *Y*_*ik*_ for person *i* in group *k* is:

$${Y_{ik}} = {b_{0k}} + {b_1}\,{\rm{Ag}}{{\rm{e}}_i} + {b_2}{X_{ik}} + {b_3}X{\prime _{ik}} + {b_4}I{\prime _{ik}} + {e_{ik}}$$

where *X*_*ik*_ is person *i*’s own popularity in group *k*, *X′*_*ik*_ is the average popularity of the other *n*–1 classmates of person *i* in group *k*, and *I′*_*ik*_ is the average similarity of the popularity of all possible pairs of classmates of person *i* in group *k.*

Fourth, to investigate whether alcohol consumption varied as a function of social comparisons between individual and peer popularity (Hypothesis 3), a *contrast model was* estimated by constraining the *actor effect* (*X*) and *others’ effect* (*X′*) to be equal in magnitude but with opposite signs (i.e., *contrast effect*, *b*_2_–*b*_3_ = 0; Kenny and Garcia 2012) and with the *others’ dissimilarity effect* (*I′*) constrained to zero. The SABIC of the *contrast model* was compared with the *complete model* to assess improvement of model fit. To confirm a social comparison process, the *contrast effect* had to be significant and the *contrast model* had to fit at least as well as the *complete model* (Garcia et al. 2015). Fifth and finally, a *contrast and others’ dissimilarity model* was estimated by including the *others’ dissimilarity effect* (*I′*) again. The SABIC of this model was compared with the *contrast model* to assess improvement in model fit after including the *others’ dissimilarity effect* again.

## Results

### Preliminary Analysis

Alcohol consumption ranged from 0 to 18 (*M*_alcohol_ = 1.44; SD_alcohol_ = 3.40); 23.9 % (*n* = 179) of the participants reported to have drank alcohol in the last 30 days. Average alcohol consumption in classrooms ranged from .00 to 5.70 indicating reasonable between-group variability and warranting further investigation of the associations between group composition and individual alcohol consumption. As expected, more adolescents drank alcohol and adolescents drank more in later grades (Grade 8: 6.8 %, *M* = .26, SD = 1.31; Grade 9: 25.4 %, *M* = 1.22, SD = 2.86; Grade 10: 41.6 %, *M* = 3.04, SD = 4.79). Alcohol consumption was positively skewed in all classrooms except one, indicating that while most adolescents drank small amounts of alcohol, a few drank more.

The average individual popularity rating received from classmates ranged from −2.32 to +2.70 (*M*_popularity_ = .81; SD_popularity_ = 1.11). Classroom averages ranged from .17 to 1.36. Individual scores were negatively skewed (−.448) meaning most participants scored positively on popularity. A one-way ANOVA of individual popularity with classrooms as a between-subjects factor showed no mean differences in individual popularity between classrooms, *F*(30, 769) = 1.408, *p* = .073, *ICC* = .015. At the individual level, popularity was moderately associated with alcohol consumption (*r* = .25, *p* < .001) showing that more popular adolescents drank more alcohol.

### Group Popularity Composition and Alcohol Use

The GAPIM results are shown in Table 1. There was a significant positive association between age and individual alcohol consumption (*b*_1_ = 1.077, *p* < .01), indicating that alcohol consumption increased with age. In the *Empty* model, 10 % of the variance in alcohol use was explained by age. The follow-up models indicated that this positive association of age decreased slightly when the group composition terms were added to the model.

**Table 1:** Group composition effect estimates of popularity on individual alcohol consumption

Model	Covariates	Main effects		Dissimilarity effects	Fit	
	Age *b*_1_	Actor popularity^a^ *b*_2_	Others’ popularity^b^ *b*_3_	Others’ dissimilarity^c^ *b*_4_	SABIC^d^	Adjusted *R* ^2^
Empty	1.077**	0^e^	0^e^	0^e^	3853.167	.103
Main effects only	.929**	.633**	−.183	0^e^	3821.739	.141
Complete	.874**	.619**	−1.046	2.229^†^	3819.795	.162
Contrast	.974**	.613** ^f^	−.613** ^f^	0^e^	3818.619	.138
Contrast and others’ dissimilarity	.844**	.636** ^f^	−.636** ^f^	2.032^†^	3816.617	.162

^†^ *p* < .10; *p < .05; **p < .01

^a^ Average received-by-classmates popularity score for each participant

^b^ Average popularity score of the others in the classroom excluding the focal participant

^c^ Standard deviation in popularity of the *n*–1 others in the classroom multiplied by −1

^d^ A significantly smaller SABIC (Sample-size-Adjusted Bayesian Information Criterion) means a better fitting model

^e^ Constrained to zero

^f^ Constrained to be equal to each other but with opposite signs (*b*_1_−*b*_2_ = 0)

In the *complete model*, individual drinking was significantly and positively associated with *actor popularity* (*b*_2_ = .619, *p* < .01), non-significantly and negatively associated with *others popularity* (*b*_3_ = −1.046, ns), and marginally significantly and positively associated with *others’ dissimilarity* (*b*_4_ = 2.229, *p* < .10)*.* Although the *others’ effect* was not significant, the fact that it was opposite in sign and close in magnitude to the *actor effect* may indicate a significant *contrast effect* between *actor popularity* and *others´ popularity* which was modeled in the follow-up *contrast* submodel.

Following Garcia et al.’s (2015) recommendations, the best submodel was selected based on the following criteria: (a) The best submodel should fit at least as well as the *complete model* (the model including all three GAPIM terms), (b) the term added to the submodel should be significant, and (c) it should be the best fitting submodel (have the lowest SABIC) of all submodels that have significant estimates of their submodel terms. Compared to the *complete model* and the *contrast model*, the fit of the final *contrast and others’ dissimilarity model* was better (had a smaller SABIC) and the terms added in this model were significant. Hence, the *contrast and others’ dissimilarity model* will be interpreted. Compared to the *empty model* (age only), including the group composition terms in this final model explained an additional 5.9 % of the variance.

The group composition estimates in the final best-fitting model (*contrast and others’ dissimilarity model*) were as follows. There was a significant and positive association between *actor popularity* and individual drinking (*b*_2_ = .636, *p* < .01) and a significant and negative association between *others’ popularity* and individual drinking (*b*_3_ = −.636, *p* < .01). Since these associations with *actor popularity* and *others´ popularity* were constrained to be equal but with opposite signs (i.e., *contrast effect*), they should be interpreted jointly. This means that more popular adolescents drank more when their classmates were, on average, less popular. Finally, there was a marginally significant association of *others’ dissimilarity* with individual drinking (*b*_4_ = 2.032, *p* < .10). This indicated that adolescents consumed less alcohol when there was more heterogeneity in popularity among their classmates (i.e., when their classmates were more dissimilar in popularity to each other).

**Fig. 1:**
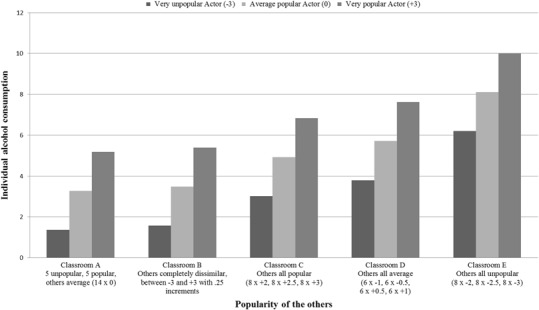
Predicted means of popularity group composition effects on adolescent alcohol consumption (*M*_age_ = 14.73) for 25-person groups with different compositions (e.g., Group B contains 8 others who score +2 on popularity, 8 score +2.5, and the remaining 8 score +3)

To further interpret these associations, we computed the predicted means for various combinations of predictors as recommended by Kenny and Garcia (2012). These predictions are shown in Fig. 1. Figure 1 shows the predicted levels of alcohol consumption of a very unpopular (−3), an average (0), and a very popular (+3) adolescent (*M*_age_ = 14.73) in five different prototypical classrooms. Each classroom consists of 25 students including the student to be influenced. Classroom A has 5 unpopular students (popularity score of −3), 5 popular students (+3), and 14 average students (0). In Classroom B all other students are completely dissimilar to each other with popularity scores ranging from −3 to +3 with .25 increments. In Classroom C all 24 classmates are popular with popularity scores of +2, +2.5 and +3 equally distributed across them (8 scoring +2, 8 +2.5, and 8 +3). In Classroom D all 24 classmates are average in popularity, having equally distributed popularity scores of −1, −.5, +.5, and +1. Finally, in Classroom E all other students are unpopular with popularity scores of −2, −2.5 and −3 equally distributed across them.

Figure 1 replicates that popular adolescents drink more alcohol than average or unpopular adolescents. Comparing the predicted means for very popular adolescents across classrooms, Fig. 1 shows that very popular adolescents drink even more when they are in a classroom of unpopular others (Group E), compared to, for example, a classroom of popular others (Group C). This illustrates the significant *contrast effect* between *actor popularity* and *others’ popularity*. Figure 1 also shows that adolescents drink less when their classmates are more dissimilar in popularity (Groups A and B) than when they are more similar in popularity (Groups C, D, and E), indicating the significant *others’ dissimilarity effect*.

## Discussion

In adolescence, alcohol consumption is associated with popularity: Popular adolescents generally drink more than less popular adolescents (e.g., Ali et al. 2014; Balsa et al. 2011; Hubers et al. 2016; Mayeux et al. 2008; Tucker et al. 2011). However, a purely individual perspective on popularity and alcohol consumption ignores the complex role that the peer group may play in adolescent drinking. Since drinking does not occur in a social vacuum and popularity is a ranking in the social hierarchy (Bukowski 2011), taking the group into account is important. Associations between popularity and alcohol consumption may vary by phenomena such as the relative difference in popularity between adolescents and their peers, and the heterogeneity in status among them (Hartup 2005). Therefore, this study combined both individual and group-level perspectives on popularity and adolescent drinking. More specifically, we investigated whether adolescent alcohol consumption was related to their own popularity, the average popularity of the others in their classroom, the contrast between adolescents’ own popularity and the average popularity of their classmates, and the heterogeneity of popularity in the classroom. Four specific hypotheses were tested.

### Individual Popularity vs. Peers’ Popularity

We hypothesized that adolescent alcohol consumption was positively associated with individual popularity (Hypothesis 1). Consistent with our hypothesis and previous research (e.g., Ali et al. 2014; Balsa et al. 2011; Hubers et al. 2016; Mayeux et al. 2008; Tucker et al. 2011), more popular adolescents drank more alcohol. However, this individual perspective tells only half the story. The relative difference in popularity between adolescents and their peers also played a role. As expected (Hypothesis 3), the association between adolescents’ own popularity and their alcohol use increased with decreasing popularity of all others in the classroom. Alcohol consumption was highest when popular adolescents were surrounded by less popular classmates.

How can we understand this finding? Drinking alcohol solicits peers’ approval, because it symbolizes adult privilege (e.g., Engels 2003). Drinking helps adolescents to become popular or maintain their popularity and avoid rejection or exclusion. Drinking alcohol also is a way for adolescents to set themselves apart from others, thereby strengthening their feelings of being the only one who is acting as an adult. Adolescents’ tendency to set themselves apart may be the strongest when they are more popular than all others around them, a social comparison process (Festinger 1954). Especially in this context, they may feel the urge to maintain their high status (Cillessen 2011). Furthermore, highly popular adolescents may fear rejection and exclusion, especially when they are much more popular than everyone else (i.e., a big tree attracts the woodsman’s axe), and this may explain why popular adolescents drink more especially among less popular others.

We also hypothesized that adolescent alcohol consumption was positively associated with the average popularity of classroom peers (Hypothesis 2). This hypothesis was not confirmed. We found no association between the average popularity in the classroom and drinking. Individual alcohol consumption was not independently related to the average level of popularity in the classroom. One explanation for this finding may be that alcohol consumption is unlikely to occur within the classroom context or on school premises. As such, knowledge about classmates’ drinking behavior or opportunities to perceive and attend to the drinking behavior of classmates may be limited, thereby limiting the influence that popular classmates may have on adolescents’ individual drinking (Brown 1990). It may also be due to the relatively young sample of this study and the fact that these adolescents do not hang out yet with their peers at bars or other establishments where they can drink alcohol. Furthermore, given that popularity is a ranking that allows adolescents to compare themselves to others (Bukowski 2011), the classroom’s average popularity may be less important than the relative difference between adolescents’ own popularity and the average popularity of their classmates.

### Heterogeneity of Popularity Among Classmates

This study also confirmed our hypothesis that alcohol consumption was lower in popular-heterogeneous groups than in popular-homogeneous groups (Hypothesis 4). More diversity in popularity among classmates was associated with less individual alcohol consumption. Alcohol consumption is perceived to be normative by adolescents at some point during their development. However, conformity to this perceived group norm (e.g., Teunissen et al. 2012) may be lower in groups that are more heterogeneous in popularity, which in turn may make adolescents in these classrooms less likely to drink.

Group identification and cohesion may be lower in popularity-heterogeneous groups due to lower perceived similarity among classmates (Hogg 1993). In less cohesive classrooms, adolescents may feel less pressure to conform to group norms (Aronson 2011; Cialdini and Trost 1998) resulting in less individual alcohol consumption. This would be consistent with the principles of self-categorization theory (Turner et al. 1987). In addition, in popularity-heterogeneous groups there may be a less salient understanding of whether and to what degree alcohol consumption is prototypical behavior that determines one’s status (Tindale et al. 2001).

### Strenghts and Limitations

Among the strengths of this study are the use of a new methodological framework that allows for the simultaneous estimation of individual and group-level effects in adolescent peer research. The Group Actor-Partner Interdependence Model (GAPIM) is a new methodological framework that so far has not been used in peer relations research, but seems very promising for it. It should also be noted that we used the GAPIM in its most simple form. Further extension of this model are possible (see Kenny and Garcia 2012). Thus, this new and innovative approach can have a large impact on further understanding the impact of peer groups on adolescent development.

Others strengths of this study were the large sample and reliable methods. The complete round-robin ratings of popularity in all classrooms were also a strength, providing for detailed assessments of peer group popularity in all classrooms.

This study also had some limitations. First, this study was cross-sectional. This means that no causal inferences can be made, such as whether popularity predicts alcohol use or whether alcohol use predicts popularity. It is also not possible to infer whether alcohol use is more beneficial in acquiring or in maintaining status. Future longitudinal studies should focus on causal patterns between group popularity composition and alcohol use. The challenge of longitudinal analyses is that they increase the complexity of the models as individual popularity and the average and variability of group popularity will also vary over time. Moreover, we expect both pathways to work concurrently which further adds to the complexity of the model. In addition, to assess how popularity and alcohol consumption co-evolve over time, the first assessment preferably needs to take place at the beginning of the school year when a new group is formed.

Second, given that the data were collected at one secondary school using a relatively young sample, we do not know whether the findings can be generalized to a broader adolescent population. Some of our findings may be related to specific characteristics of this school or to our relatively young sample. However, a very important reason to investigate this relatively young (early adolescent) sample is that this is the age when adolescents often start drinking. Moreover, at this age, being popular is perceived as highly important and peer influence and susceptibility to peer influence peak (LaFontana and Cillessen 2010; Sandstrom 2011). Given the developmental path of peer influence processes, the findings may be relatively strong in middle compared to early and late adolescence. In addition, the relatively low prevalence of alcohol use in early adolescence may have resulted into relatively weak associations between peer group popularity composition and individual alcohol use. Future studies should attempt to replicate this study with older adolescents and in other schools (e.g., with different educational tracks).

### Directions for Future Research

In the interest of model parsimony and interpretation of the associations between group composition and individual drinking, only age was included as an additional individual characteristic related to drinking. Although beyond the scope of this study, associations between group popularity composition and individual alcohol use may also vary by other individual characteristics (e.g., gender, personality traits), dyadic characteristics (e.g., a best friend’s alcohol use), or group characteristics (e.g., similarity in attitudes, values, beliefs, expectations regarding alcohol use). For example, alcohol consumption varies by gender and thus associations between group popularity composition and individual alcohol use may be moderated by gender. Future studies could address these related research questions. Another example regards alcohol-related beliefs. Previous research has shown that alcohol consumption is positively affected by cognitions about drinking such as positive alcohol expectancies and lower self-efficacy (e.g., Blume et al. 2003). Future research may expand the current study by investigating whether associations between group popularity composition and individual alcohol consumption vary as a function of individual- and group-level alcohol-related beliefs.

Another direction for future research is to expand the context in which group popularity composition is investigated. For example, it would be interesting to examine the unique associations between group popularity composition and individual drinking within the friendship network, in addition to the classroom context. Although this study and previous work clearly shows the significant influence of classmates on adolescents’ individual antisocial behaviors (e.g., Gommans et al. 2016; Müller et al. 2015), individual alcohol consumption may also be affected by comparisons of oneself to one’s close friends, in addition to one’s classmates in general. Furthermore, as alcohol use increases with age and peer networks expand beyond the classroom, individual alcohol use will also be affected by out-of-classroom and out-of-school popularity characteristics, for example, how popular one is in an after-school peer group and how popular these out-of-school peers are.

A final issue that deserves further study is whether the *contrast effect* between adolescent and group popularity on individual alcohol consumption might be curvilinear. It may be that the effect holds up to a certain point, but diminish once the difference in popularity between adolescent and group becomes too large. For example, in a group in which an adolescent is much more popular than everyone else, alcohol consumption may no longer be a salient characteristic helping the adolescent to maintain their popularity. A moderate difference in popularity between an adolescent and all other group members is likely to solicit more individual alcohol consumption than a smaller or larger difference (van Knippenberg and Schippers 2007).

## Conclusion

This study demonstrated that individual alcohol consumption depends not only on adolescents’ own popularity in the classroom, but also on the relative difference in popularity between adolescents and the average popularity of all others in the classroom. More popular adolescents drink more alcohol, but even more so when they are themselves more popular than the average popularity of their classmates. Furthermore, this study showed a reduction of alcohol use with increasing heterogeneity in popularity in the classroom. Conformity pressures to use alcohol then may be lower and alcohol consumption then may be less a prototypical behavior that determines one’s position in the status hierarchy. Together, the results of this study emphasize that to fully understand adolescent drinking, research should not only examine individual characteristics such as status but also the characteristics of the groups adolescents are interacting in, and importantly the interaction between individual and group characteristics. This principle may also be extended to the study of emergence of other antisocial and health risk behaviors in adolescent peer groups.

## Footnotes

1 We use the word “effect” in accordance with existing methodological terminology. Since we used cross-sectional data, it is important to note that the term effect in this research only implies association, not causation.
